# Mechanical cues as immunomodulators in neuroinflammation-driven spinal sensitization: analgesic mechanisms and therapeutic strategies

**DOI:** 10.3389/fimmu.2026.1791900

**Published:** 2026-04-30

**Authors:** Li-ya Tang, Ke-shang Li, Xiao-xia Kuang, Jiang-shan Li, Xiang Feng, Wu Li

**Affiliations:** 1 College of Acupuncture, Massage and Rehabilitation, Hunan University of Chinese Medicine, Changsha, Hunan, China; 2 The Second Affiliated Hospital of University of South China, Hengyang, Hunan, China

**Keywords:** analgesia, immunoregulation, mechanical stimulation, neuroinflammation, spinal sensitization

## Abstract

Neuroinflammation is a key immunological driver of spinal sensitization and the transition from acute to chronic pain. Although mechanical interventions can reduce pain, the immuno-inflammatory mechanisms linking mechanical cues to resolution of spinal neuroinflammation remain poorly integrated and inconsistently defined. A major gap is the lack of a mechanobiological framework that connects mechanotransduction with glial activation, cytokine/chemokine signaling, and neuroimmune synaptic plasticity, and translates these mechanisms into measurable biomarkers and reproducible protocols. Here, we synthesize preclinical and clinical evidence showing how mechanical stimuli may modulate neuroinflammation-driven sensitization. We summarize mechanotransduction pathways that shape microglial and astrocytic reactivity, inflammatory signaling, and downstream remodeling of ion channels, receptors, and synaptic circuits. We also discuss how mechanical interventions may shift the spinal inflammatory microenvironment by improving perfusion and metabolic homeostasis. Finally, we outline candidate biomarker panels and highlight key limitations, including heterogeneity in dosing, outcomes, and translational models. Overall, mechanical forces may act as immunomodulatory cues that reprogram neuroinflammation and weaken spinal sensitization, supporting a mechanistic basis for non-pharmacological analgesia. Future progress requires standardized mechanical dosing, mechanism-informed biomarkers, and rigorous translational pipelines to enable quantifiable, personalized mechanotherapy for chronic pain.

## 1 Introduction

Chronic pain represents a major global public health challenge, affecting a substantial proportion of the adult population. It is estimated that approximately 30% of the world’s population suffers from chronic pain (1), Among them, about 14% are diagnosed with other serious comorbid conditions, resulting in considerable socioeconomic burden and psychosocial impairment (2). Chronic pain is frequently underestimated and undertreated, leading to functional limitations, reduced quality of life, and increased healthcare expenditures (3). At present, clinical pain management remains heavily dependent on pharmacological treatments, including nonsteroidal anti-inflammatory drugs, anticonvulsants, and opioids (4). Although multiple pharmacological options are available, their long-term use is often limited by modest efficacy, prominent adverse effects, and the risk of dependence or misuse (5), These limitations have stimulated increasing interest in mechanism-based non-pharmacological intervention strategies.

Over the past two decades, studies have demonstrated that spinal sensitization driven by neuroinflammation represents a key pathophysiological substrate for the transition from acute to chronic pain (6, 7). Activation of glial cells and immune-related signaling in the peripheral and central nervous systems leads to the release of pro-inflammatory mediators, which in turn trigger neuroinflammation and amplify nociceptive transmission (8). Spinal sensitization, characterized by increased neuronal excitability and altered synaptic plasticity in the dorsal horn of the spinal cord, underlies hyperalgesia and mechanically evoked allodynia in various pain conditions (9). Therefore, a comprehensive understanding of the mechanisms governing the initiation and persistence of neuroinflammation-driven spinal sensitization is critical for the development of targeted non-pharmacological analgesic interventions.

Manual physical pressure (tuina) is a commonly used manual therapy in traditional Chinese medicine and is widely used for the management of spine-related pain and peripheral neuropathic pain (10). From a modern biomedical perspective, tuina can be regarded as a form of controlled mechanical stimulation applied to the body surface, muscles, fascia, and paraspinal structures (11). This mechanical input modulates nociceptive processing at both local and central levels. Existing clinical and preclinical studies suggest that the analgesic effects of tuina are, to some extent, comparable to those of other manual therapies, however, its underlying cellular and molecular mechanisms remain incompletely understood (12)。Re-examining tuina within the theoretical framework of mechanobiology may help bridge traditional practice and contemporary pain science.

Given accumulating evidence that mechanical force–based stimulation can modulate neuroinflammation and spinal sensitization, tuina has the potential to serve as an important model for elucidating the mechanisms of non-pharmacological analgesia. This review first outlines the pathophysiological basis of neuroinflammation and spinal sensitization in chronic pain. It then summarizes the regulatory effects of mechanical stimuli, particularly tuina, on these processes at the cellular and molecular levels. It integrates findings from both preclinical and clinical studies, discusses methodological considerations, and proposes a mechanobiology-based framework for understanding the analgesic mechanisms of tuina. In addition, it identifies key knowledge gaps in current research and outlines future directions to advance the translational and personalized application of tuina in pain management.

## 2 Pathophysiology of spinal sensitization driven by nerve inflammation in pain

**Figure 1:**
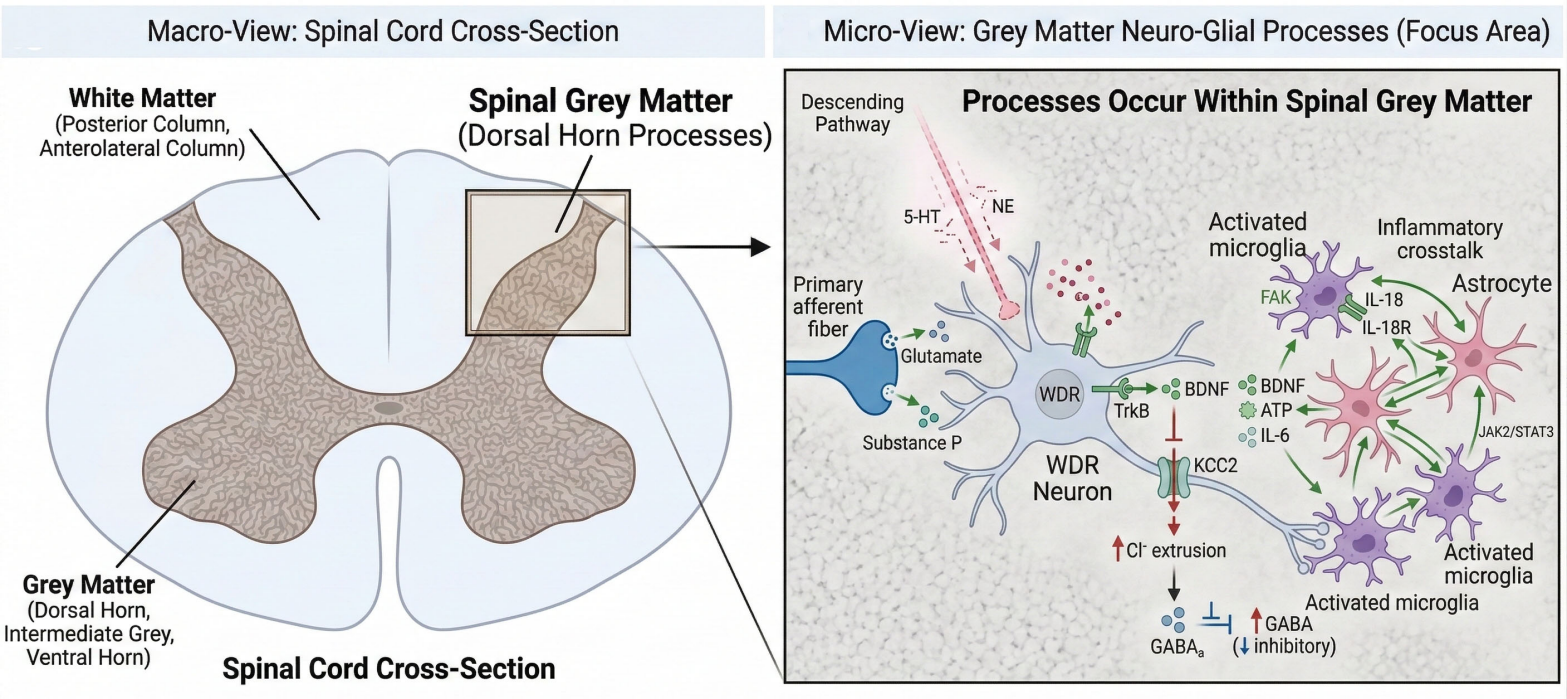
A framework of cellular and molecular mechanisms of spinal sensitization driven by neuroinflammation in chronic pain.

The pathophysiological basis of neuroinflammation-driven spinal sensitization in the context of pain involves glial cell activation, release of inflammatory mediators, altered signaling pathways, and central sensitization within the spinal cord (see **Figure 1**). Understanding these processes provides a theoretical foundation for developing non-pharmacological analgesic strategies that target neuroimmune pathways.

### 2.1 Overview of neuropathic, nociceptive and nociplastic pain

Pain is a multidimensional and complex subjective experience that can be broadly classified into nociceptive, nociplastic, and neuropathic pain, each with relatively specific yet partially overlapping pathophysiological mechanisms (13). Nociceptive pain arises from noxious stimuli acting on otherwise normal tissues (14), whereas nociplastic pain results from the release of inflammatory mediators following tissue injury, such as cytokines that contribute to arthritic pain (15). Neuropathic pain originates from lesions or diseases of the nervous system, including nerve compression and spinal cord injury, and is often accompanied by sensory abnormalities and refractory pain (16). For example, persistent neuropathic pain may occur after spinal cord injury and is associated with abnormal accumulation of ATP at the lesion site, which leads to microglial activation (17). In models of nociplastic pain, factors such as IL-1β induce hyperexcitability and nociceptive sensitization within the spinal cord (18). Neuropathic pain results from nerve injury, involving hyperactive neurons, activated microglia and astrocytes, and upregulated mechanosensitive channels (Piezo2, TRPV1, P2X3/4), driving central sensitization and chronic hyperalgesia (19). In contrast, nociceptive pain is triggered by tissue injury, mediated by peripheral nociceptors and transient inflammatory mediators, with limited central plasticity (20). Clinically, neuropathic pain is often burning or electric-like, while nociceptive pain is localized and aching (21). Together, these processes contribute to persistent hyperalgesia and mechanically evoked allodynia in clinical settings (22). Clinically, most chronic pain conditions present as mixed pain phenotypes and are accompanied by maladaptive plasticity within the spinal cord and brain.

### 2.2 Cellular components of neuroinflammation in the spinal cord

Neuroinflammation within the spinal cord entails coordinated contributions from multiple cell types, including microglia, astrocytes, oligodendrocytes, and infiltrating peripheral immune cells. Under pain conditions, glial cells in the dorsal horn of the spinal cord—primarily microglia and astrocytes-represent the principal effector population mediating neuroinflammation. Microglia are activated following nerve injury and drive inflammatory responses by releasing pro-inflammatory cytokines and other mediators, such as TNF-α, IL-1β, and TLR4. They also participate in synaptic pruning and neuronal hypersensitization, thereby contributing to the initiation and maintenance of neuropathic pain (23). For example, in chronic pain models, proliferation of spinal microglia and upregulation of ionized calcium-binding adaptor molecule 1 (Iba-1) have been observed, accompanied by reduced pain thresholds (24). Astrocytes contribute to chronic pain mechanisms by regulating glutamate uptake and releasing pronociceptive substances, such as ATP and various gliotransmitters (25). Dysregulation of astrocytic glutamate uptake has been reported to facilitate nociceptive signal transmission (26). In addition, peripheral immune cells, such as macrophages and T cells, can infiltrate the dorsal root ganglia (DRG) and spinal cord after nerve injury, thereby amplifying inflammatory responses. Macrophages comprise both resident and infiltrating subpopulations in the spinal cord. For instance, MRC1-positive spinal macrophages proliferate after nerve injury and exert anti-inflammatory effects by suppressing glial activation, thereby limiting neuroinflammation and promoting pain resolution (27). Regulatory T cells are peripheral immune cells that infiltrate the spinal cord and alleviate neuroinflammation and pain by inhibiting microglial activation and pyroptosis (28). In summary, a cellular network composed of neurons, glial cells, and peripheral immune cells drives pain-related neuroinflammation through reciprocal interactions, thereby amplifying neuro–immune crosstalk and playing a crucial role in chronic pain.

### 2.3 Molecular mediators and signaling pathways

Neuroinflammatory mediators and signaling pathways are largely orchestrated by astrocytes and microglia, which amplify pain signaling through synaptic modulation and neuroinflammatory cascades. Aberrant activation of the JAK/STAT, NF-κB, MAPK (ERK, p38, JNK), and TLR4/MyD88 pathways represents a core mechanism of neuroinflammation (29–32). In addition, upregulation of the Wnt/β-catenin pathway and mechanosensitive channels (TRPV1, TRPA1, P2X4/7) enhances the release of pro-inflammatory cytokines and neuronal excitability, thereby mediating neuroinflammation (33). This pathway is upregulated in astrocytes and, through interactions with microglia, promotes the release of pro-inflammatory cytokines such as IL-6 and IL-18, while simultaneously suppressing the anti-inflammatory cytokine IL-10 (34). Following nerve injury, as in models of chronic constriction injury of the sciatic nerve, cerebrospinal fluid levels of IL-6 are markedly increased, triggering STAT3 activation in astrocytes (35). This activation facilitates the spread of neuroinflammation and contributes to the development of widespread pain. The IL-18/IL-18R axis mediates crosstalk between microglia and astrocytes, driving cytokine release and inflammatory cascades and thereby exacerbating pain hypersensitivity. Specifically, IL-18 released from microglia binds to IL-18 receptors (IL-18R) on astrocytes, thereby activating downstream inflammatory responses that contribute to pain generation (36). In addition, the Wnt signaling pathway is abnormally activated in the sciatic nerve, dorsal root ganglia, and dorsal horn of the spinal cord. This signaling promotes activation of microglia and astrocytes and the release of pro-inflammatory mediators, leading to mechanical hyperalgesia (37). Other microglia-derived inflammatory mediators, such as tumor necrosis factor-α and interleukin-1β, can enhance synaptic transmission, resulting in central sensitization and pain hypersensitivity. In nerve injury models, these mediators drive neuroinflammation and promote the progression of neuropathic pain (38, 39).

In addition to classical inflammatory pathways, adenosinergic and Ca²^+^ signaling, as well as potassium channel-related pathways, regulate intracellular signaling cascades and intercellular communication, thereby directly influencing the pathophysiological progression of neuroinflammation (40, 41). In trigeminal neuralgia models, β-fiber stimulation activates adenosinergic mechanisms in astrocytes and, via ATP signaling mediated by P2X4/P2X7 receptors on glial cells, induces neuroinflammation and amplifies nociceptive signaling (42). In the rostral ventromedial medulla (RVM), astrocyte-dependent Ca²^+^ signaling has been shown to exert analgesic effects (43). In addition, the inwardly rectifying potassium channel Kir4.1 in astrocytes regulates cellular excitability and neuroinflammation, and its dysfunction contributes to the development of chronic pain (44). Overall, the interplay among cytokine networks, receptor and ion channel activation, and downstream signaling cascades constitutes the molecular basis of neuroinflammation under pain conditions.

### 2.4 Mechanisms of spinal sensitization

Spinal sensitization (central sensitization) refers to a state in which the excitability and synaptic efficacy of neurons in the dorsal horn of the spinal cord are enhanced under persistent nociceptive input, leading to reduced pain thresholds and amplified pain perception. Its mechanisms include the following five aspects:

#### 2.4.1 Long-term potentiation-like synaptic changes

Intense peripheral input can increase the firing frequency of wide dynamic range (WDR) neurons in the dorsal horn and reorganize their firing synchrony (45). For example, repeated mechanical stimulation can induce segmental and extrasegmental temporal summation of pain (“wind-up”), indicating enhanced central synaptic efficacy (46).

#### 2.4.2 Increased release of excitatory neurotransmitters

Injury or inflammation causes primary afferent neurons to release excessive glutamate and substance P, which activate NMDA receptors and neurokinin receptors, thereby producing sustained excitation of dorsal horn neurons (47). In addition, studies have shown that the spinal NR2B-containing NMDA receptor-Ca²^+^/calmodulin-dependent protein kinase II (CaMKII) pathway is involved in sensitization in nociplastic pain (48).

#### 2.4.3 Reduced function of inhibitory pathways

In chronic pain states, spinal GABAergic and glycinergic inhibition is diminished, leading to “disinhibitory gaps” (49). Inflammatory stimuli can reduce spinal GABA content or impair GABA receptor function, thereby weakening the inhibitory control of nociceptive signals (50). At the same time, the function of descending inhibitory pathways originating from the periaqueductal gray and medullary structures is compromised, and insufficient release of endogenous analgesic mediators such as 5-HT and noradrenaline further aggravates central sensitization (51, 52).

#### 2.4.4 Neuron–glia interactions

Factors released from glial cells, such as brain-derived neurotrophic factor (BDNF), can increase the excitability of nociceptive relay neurons in the spinal cord (53). For instance, reduced Cl^-^ influx can lead to a functional switch of GABAergic transmission from inhibition to excitation, thereby promoting the formation of central sensitization (54).

#### 2.4.5 Network and structural remodeling

Chronic pain is accompanied by structural changes in spinal circuits. One example is sprouting and synaptic reorganization of Aβ fibers, whereby low-threshold mechanoreceptive fibers form aberrant connections with nociceptive pathways, causing tactile stimuli to be perceived as painful (allodynia) (55).

In summary, enhanced synaptic plasticity, loss of inhibition, and glia-mediated inflammatory amplification together shape the pathophysiological basis of spinal sensitization.

## 3 Mechanical stimulation and mechanotransduction in the peripheral sensory-spinal pathway

**Figure 2:**
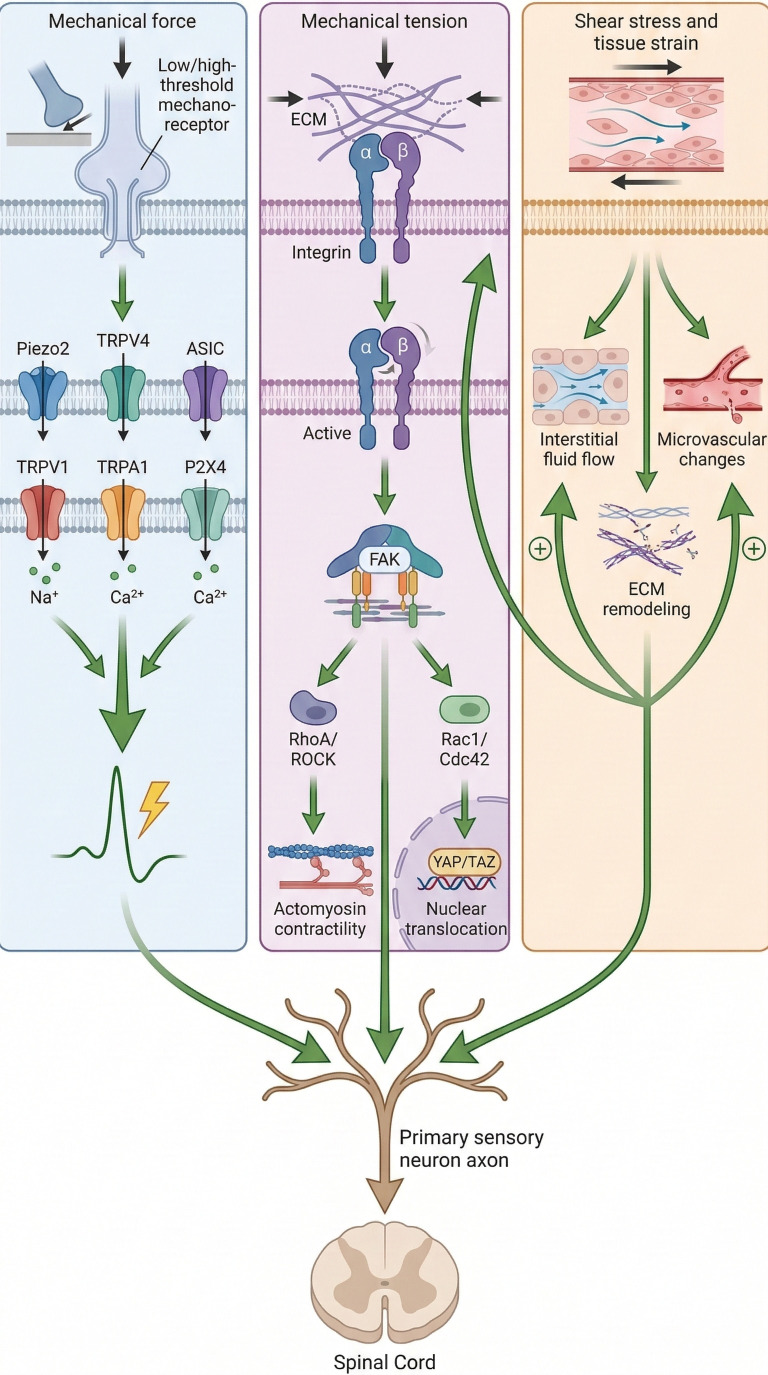
Multilevel force biology pathway of mechanical stimulation.

Mechanical stimulation modulates neuroinflammation and pain sensitization along the peripheral sensory-spinal pathway, including peripheral afferents, dorsal root ganglia, spinal dorsal horn neurons, and glial cells. (see **Figure 2**).

### 3.1 Biomechanical characteristics of mechanical stimulation

Manual therapies such as tuina apply mechanical forces to the body through techniques including pressing, kneading, traction, and twisting. The biomechanical characteristics of these techniques are defined by parameters such as force magnitude (pressure/tension), frequency, and duration (56). In a study of patients with temporomandibular joint pain, two levels of repetitive mechanical stimulation (0.5 kg and 1.0 kg) were applied, and pain sensitization was assessed. The results showed that the affected joints on the painful side exhibited significantly greater mechanical sensitivity and a more pronounced wind-up (temporal summation) effect than the contralateral side and healthy controls (57, 58), suggesting that the intensity and frequency of mechanical stimulation influence pain responses. In general, low-frequency, high-amplitude pressing induces deformation and stretching of deep tissues, whereas high-frequency, small-amplitude vibration predominantly stimulates superficial sensory receptors. From a biomechanical perspective, tuina may improve the local mechanical environment, for example, by modulating muscle tone and fascial adhesion and enhance local microcirculation and tissue elasticity (59). Quantitative studies of these mechanical effects remain limited and lack unified standards; however, preliminary attempts have been made to quantify the force and frequency of manual techniques using force sensors and related devices (60). Overall, the magnitude and pattern of mechanical loading in tuina procedures determine how forces are distributed within tissues, with different manipulations likely targeting distinct receptors and producing divergent biological effects.

### 3.2 Peripheral mechanoreceptors and mechanosensitive ion channels

The body converts external mechanical stimuli into neural signals through mechanoreceptors, a process that primarily depends on mechanosensitive ion channels. These channels are activated by mechanical forces and mediate transmembrane ionic currents, thereby transducing mechanical stimuli into electrochemical signals (61). The peripheral terminals of primary sensory neurons in the skin, muscles, and joints contain a variety of mechanosensitive structures. Low-threshold mechanoreceptors (such as Meissner’s corpuscles and Pacinian corpuscles) detect touch and vibration, whereas high-threshold mechanonociceptors detect strong, noxious mechanical stimuli. Mechanosensitive ion channels expressed on these receptors constitute the molecular basis of mechanotransduction (62).For example, Piezo2 channels mediate the rapid transduction of touch and proprioceptive signals (63). TRPV4 channels are sensitive to tissue stretch and osmotic changes and contribute to inflammation-related mechanical pain (64). Acid-sensing ion channels (ASICs) have also been implicated in pain induced by mechanical stimulation (65). Notably, noxious mechanical stimulation is often accompanied by the release of inflammatory mediators, and polymodal ion channels such as TRPV1 and TRPA1 can be activated and contribute to mechanical pain signaling (66). TRPA1 has been identified as a key mediator of pain induced by metabolic by-products; for instance, the glycolytic by-product methylglyoxal can trigger pain and spinal neuronal excitation via TRPA1 (67). In addition, although P2X4 receptors primarily respond to ATP, P2X4 expressed on spinal glial cells is also regarded as an important mediator of mechanical neuropathic pain, because tissue injury is commonly associated with ATP release (68). In summary, a sequence of events involving mechanical stimulation, deformation of peripheral receptors, and opening of ion channels converts mechanical forces into neural impulses. Different types of mechanical stimuli engage distinct ion channels and fiber populations, thereby shaping subsequent pain transmission.

### 3.3 Mechanotransduction pathways: integrins, cytoskeleton, and downstream signaling

Integrins are key mechanosensory receptors. As transmembrane receptors, integrins possess extracellular domains that sense the mechanical properties of the extracellular matrix (ECM) and intracellular domains that are linked to the actin cytoskeleton via non-covalent interactions (69). When mechanical forces act on cells, ECM-bound integrins undergo conformational changes and assemble into focal adhesions, which serve as central sites of mechanotransduction. Through the actomyosin network, these focal adhesions mediate mechanical force transmission and sensing (70). The actin cytoskeleton functions as a scaffold for force transmission, and its dynamic coupling with integrins triggers cytoskeletal reorganization, thereby conveying tension to intracellular structures (71). Studies have shown that β4 integrins can indirectly regulate the cytoskeleton via their cytoplasmic tails (72). In addition, focal adhesion–associated proteins such as PEAK1 have been identified as integrin-interacting proteins that participate in signaling downstream of RGD-binding integrins (69). When tissues are exposed to mechanical stress, integrins physically link the cytoskeleton to the extracellular matrix and, through force-induced conformational changes, initiate intracellular signaling cascades (73). These cascades include Rho GTPase signaling, activation of focal adhesion kinase (FAK)/RhoA/ROCK pathways, and mechanosensitive nuclear translocation of the transcriptional coactivators YAP/TAZ. Specifically, integrin-mediated mechanical signaling activates the Rho family GTPases Rac1 and Cdc42, thereby regulating actin remodeling and cell migration (74). At the same time, integrin-dependent tension can modulate transcription factor activity, thereby influencing cell fate decisions, including differentiation and proliferation (75). Under pathological conditions, downregulation of the cytoskeletal protein CKAP4 disrupts the linkage between integrins and the microtubule cytoskeleton, leading to impaired mechanotransduction and functional abnormalities (76). Taken together, the integrin–cytoskeleton system constitutes a central hub of mechanotransduction, dynamically integrating mechanical cues at focal adhesions, activating multilevel downstream signaling networks, and ultimately regulating cellular behavior and tissue homeostasis.

### 3.4 Effects of mechanical stimulation on local microenvironment

In addition to directly modulating cellular mechanotransduction, mechanical stimulation can profoundly alter the local tissue microenvironment, including microcirculation, interstitial fluid flow, and the architecture of the extracellular matrix (ECM). In nerve injury models, abnormalities in microvessel behavior—such as dynamic displacement, increased vascular density, and pericyte proliferation—have been shown to trigger spontaneous pain and clustered neuronal discharges (77). Myogenic vascular responses induced by mechanical stimulation can further exacerbate pain. The interstitial fluid pressure gradient is a key determinant of interstitial fluid flow, and mechanical stimuli such as shear stress can modulate flow velocity and influence cellular signaling pathways, thereby activating nociceptors and driving mechanically evoked pain (78).

Moreover, mechanical stimulation shapes the onset and progression of pain by regulating ECM synthesis and repair. Studies have shown that, after nerve injury, mechanical stimulation can modulate mesenchymal stem cell differentiation, ECM deposition, and angiogenesis, thereby contributing to synaptic plasticity and pain regulation (79). Following tissue injury, neutrophils can also enhance tissue mechanical properties by forming ECM “barrier rings,” which help reduce wound-induced pain (80). These microenvironmental alterations are bidirectionally linked to neuroinflammation and may help attenuate persistent neuroinflammatory burden and reduce peripheral pronociceptive input to the spinal cord.

## 4 Effects of mechanical stimulation on neuroinflammation

**Figure 3:**
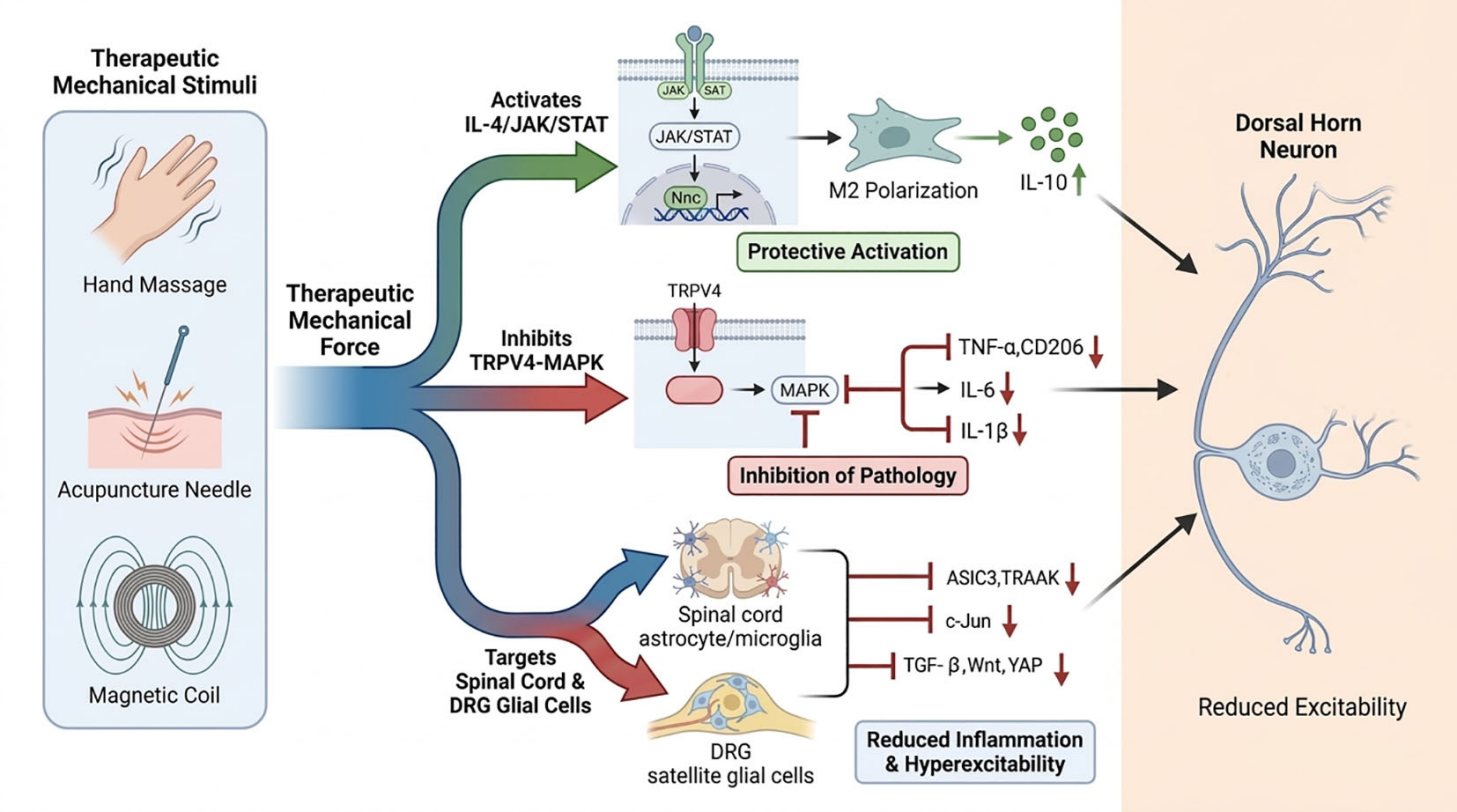
Mechanical stimulation of the central and peripheral nerve inflammation axis.

Mechanical stimulation regulates neuroinflammation bidirectionally through three core processes-mechanical signal sensing, intracellular signal transduction, and modulation of immune cell function (**Figure 3**). Based on these mechanisms, the development of mechanopharmacology-based intervention strategies holds promise as a novel therapeutic approach for neuroinflammation-related diseases. Acupuncture, massage (tuina), ultrasound, and exercise are all forms of mechanical stimulation. However, the mechanical signals they generate differ markedly in type, intensity, frequency, and target tissue. Based on these characteristics, mechanical stimuli are classified into three categories: static pressure, shear flow, and high-frequency vibration. Specifically, massage (tuina) is classified as a shear flow intervention (81), acupuncture as static pressure (82), and ultrasound therapy as high-frequency vibration (83), Exercise-based interventions may include both static pressure and shear flow components, depending on the modality (84). Despite this scientifically grounded classification, mechanical stimulation rarely produces a single, isolated effect in clinical practice. Instead, multiple mechanical effects often occur simultaneously. Furthermore, inter-operator variability and differences in practitioner experience result in inconsistencies in intervention intensity, frequency, and other parameters. This presents a significant challenge for widespread clinical application and underscores the lack of standardized guidelines for mechanical stimulation parameters.

### 4.1 Evidence from preclinical studies: animal models of pain

Mechanical stimulation therapies can suppress neuroinflammatory responses in pain states (see **Table 1**). Neuroinflammation induced by lumbar disc herniation (LDH) is a key driver of neuropathic pain, and tuina (5 N, 10 min/day, for 18 days) alleviates symptoms by reducing inflammatory responses (85). Across multiple chronic pain models, interventions such as tuina consistently reduce inflammatory mediator levels and improve pain-related behaviors. Specifically, in a rat model of knee osteoarthritis (KOA), tuina (3–5 N, 10 min/day, for 10 days) reduced pain, improved joint function, and attenuated cartilage degeneration by activating the PI3K/AKT/mTOR pathway (86). In a rat model of sciatic nerve transection and anastomosis, a tuina (0.45 N, 10 min/day, for 28 days) applied to the gastrocnemius enhanced somatosensory-evoked potential amplitudes, promoted adaptive remodeling of the somatosensory cortex, and accelerated nerve repair (87). Beyond tuina, electroacupuncture (100 Hz, 20 min/day, for 18 days) applied to acupoints improved behavioral deficits in a mouse model of autism spectrum disorder and suppressed neuroinflammatory markers in the cerebral cortex and hippocampus (88). Another related physical neuromodulation approach, noninvasive transspinal magnetic stimulation, alleviated mechanical allodynia and reduced spinal neuroinflammation in neuropathic pain rats by upregulating SOCS3 in spinal microglia (89). These findings provide a foundation for identifying the cellular and molecular targets of mechanical stimulation.

**Table 1:** Anti-inflammatory and analgesic effects of mechanical stimulation in chronic pain animal models.

Animal model	Form of intervention	Mechanical parameters	Primary target	Effect indicator	Ref.
Rat-lumbar disc herniation	Tuina	5 Newtons pressure, 10 min/day, for 18 days	Regulating downstream inflammatory cytokines through the MAP4K4/NF-κB pathway	Reduce inflammation to relieve pain	(85)
Rat-osteoarthritis	Tuina	3–5 Newton pressure, 10 min/day, for 10 days	Activating PI3K/AKT/mTOR pathway	Relieves pain, improves joint function, and reduces cartilage degeneration	(86)
Rat-anastomosis of sciatic nerve rupture	Tuina	0.45 Newtons pressure, 10 min/day, for 28 days	Regional ALFF values in the resting state of PNI rats	Promoting the adaptive changes of somatosensory cortex to achieve the recovery of peripheral nerve injury and repair	(87)
Mouse-chronic constriction injury	Tuina	4 Newton pressure, 9 min/day, for 14 days	Downregulation of TRPV4-CaMKII/CREB/NLRP3 signaling pathway	Reducing mechanical abnormal pain and hyperalgesia in CCI rats and exerting protective effects on DRG neurons	(90)
Rat-osteoarthritis	Tuina	4 Newtons pressure, 10 min/day, for 14 days	Reducing inflammation infiltration in synovial tissue and decreasing expression of TNF-α	Reducing synovial inflammation and delaying chondrocyte apoptosis	(91)
Rat-sciatica relief	Mechanical vibration	100 Hz mechanical vibration, 60 minutes/day, for 96 days	NA	Improving sensory reorganization and body awareness	(92)
Rat-mechanical pain	spinal cord stimulation	2Hz mechanical vibration, 1 min/day, 10 days	Reducing hyperactivity in pain-related brain regions	Reducing mechanical abnormal pain	(93)
Rat-mechanical pain	Acupuncture	1 mA electroacupuncture, 15 min/day, 7 days	Increasing the basal pain threshold	Reducing mechanical pain induced by high-intensity stimulation	(94)
Rat-osteoarthritis	Acupuncture	0.2 mA electroacupuncture, 15min/day, for 240 days	NF-κB pathway	Significantly reduced pro-inflammatory cytokines and cartilage degradation biomarkers	(95)
nociplastic pain	Laser acupuncture	20 mA electroacupuncture, 30 min/day.	Opioid and non-opioid pathways	Overstimulation of A-δ myelinated fibers activates the pain regulation system	(96)

### 4.2 Modulation of glial cell activation

Balancing glial cell activation is crucial for treating neuroinflammation. Mechanical stimulation modulates glial activation bidirectionally by suppressing pro-inflammatory cytokines like TNF-α and IL-1β, while also enhancing neuroprotective functions (97). Studies show that after tuina (0.45 N, 10 min/day, for 28 days) intervention, the amplitude of electrophysiological signals in the injured limbs of rats significantly increases, indicating that coordinated regulation of neuronal and astrocytic activity promotes neural recovery (87). Similarly, in acupuncture therapy (50 Hz, 20 s/day, for 14 days), needling activates neuronal and astrocytic calcium signaling in the somatosensory cortex, thus influencing neuroinflammation (98). In traumatic brain injury, interlimb mechanical stimulation (IMMA) reduces glial activation in the trigeminal ganglion and spinal trigeminal nucleus, decreases neuroinflammation and endocannabinoid dysregulation, and alleviates mechanical hypersensitivity (99). Moreover, mechanical stimulation modulates neuroinflammation by targeting mechanosensitive pathways in astrocytes (100). For example, Magnetomechanical stimulation (MMS), which uses magnetic fields to drive antibody-functionalized magnetic beads and thereby generate localized mechanical forces, can activate mechanogated channels in astrocytes and modulate neuroinflammatory signaling (101). These findings collectively highlight glial cells as central targets of mechanical therapies for modulating neuroinflammation.

### 4.3 Regulation of inflammatory cytokines and signaling pathways

Mechanical stimulation can bidirectionally regulate both the expression of pro-inflammatory and anti-inflammatory cytokines and the activation of key signaling pathways. Studies suggest that mechanical stimulation activates the IL-4/JAK/STAT signaling pathway, promoting macrophage polarization towards the anti-inflammatory M2 phenotype, reducing pro-inflammatory cytokine release (e.g., IL-1β, IL-6), and increasing IL-10 expression (102). Mechanical strain, in conjunction with TRPV4 channel inhibition, significantly reduces MAPK expression, suppresses pro-inflammatory genes (e.g., TNF-α, IL-6), and upregulates the anti-inflammatory marker CD206 expression (103). Excessive mechanical load can induce cartilage degradation, increase pro-inflammatory cytokines (e.g., IL-1β, IL-6, TNF-α), and trigger inflammatory responses (104). In addition to classical inflammatory pathways, moderate mechanical stimulation activates TGF-β signaling, increasing active TGF-β1 release, promoting cell migration, and aiding tissue repair. However, blocking TGF-β receptors or impairing primary cilia function (e.g., IFT88 deletion) inhibits this effect (105). Mechanical stimulation also activates phosphorylated YAP protein, regulating inflammation and tissue repair (106). Studies show that during neural differentiation, mechanical-electrical stimulation regulates glial cell and neuron differentiation through the Shh/Gli1 and JAK/Stat3 pathways, respectively, enhancing neurite growth and synaptic maturation (107). These results suggest that mechanical stimulation bidirectionally regulates the expression of pro-inflammatory and anti-inflammatory cytokines by activating or inhibiting signaling pathways, such as IL-4/JAK/STAT, TRPV4-MAPK, Wnt, TGF-β, and YAP. Its effects depend heavily on the precise regulation of mechanical parameters, making it a key strategy for targeted interventions in inflammation and tissue repair.

### 4.4 Neuroimmune interactions within the spinal cord and DRG

Mechanical therapies affect not only the spinal cord but also the pathological processes in peripheral ganglia. Repetitive transcranial magnetic stimulation (rTMS), a type of mechanical therapy, reduces pro-inflammatory cytokine expression (e.g., IL-1β, IL-6, TNF-α) in the dorsal root ganglion, while increasing anti-inflammatory cytokines like IL-10, thereby alleviating neuroinflammation and neuropathic pain (108). In mechanical pain due to osteoarthritic changes, tuina alters the expression of mechanosensitive ion channels in dorsal root ganglion neurons, such as acid-sensing ion channel 3 (ASIC3) and TWIK-related arachidonic acid-activated potassium channels (TRAAK). Additionally, mechanical hypersensitivity is closely linked to the activation of the TrkA-specific signaling pathway, c-Jun, in the ganglion (109). Satellite glial cells (SGCs) in peripheral ganglia, including the dorsal root and trigeminal ganglia, are activated after nerve injury or inflammation, increasing neuronal excitability and promoting chronic pain (110). Mechanical therapy regulates SGC activity through mechanical signaling, improving the ganglionic microenvironment’s homeostasis and reducing their pathological activation (111). Peripheral nerve injury (PNI) induces ganglionic lesions, with macrophages recruited to peripheral tissues like the sciatic nerve and dorsal root ganglion, and increased circulating pro-inflammatory cytokines, such as IL-6 and TNF-α (112). Mechanical therapy inhibits the abnormal infiltration of immune cells and the inflammatory cascade by modulating the mechanical microenvironment (113). In summary, mechanical interventions bidirectionally regulate neuroimmune interactions through the spinal cord and peripheral ganglia, calming spinal glial inflammation, improving the ganglionic environment, and reducing the generation and transmission of noxious impulses to achieve analgesic effects.

## 5 Effects of mechanical stimulation on spinal sensitization and neural plasticity

**Figure 4:**
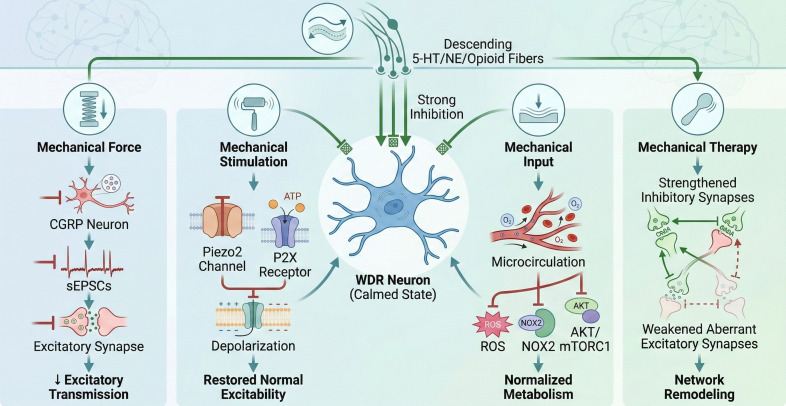
Neural plasticity effects of mechanical force stimulation in dorsal horn of spinal cord.

Importantly, the analgesic effects of mechanical stimulation on spinal plasticity are closely linked to its immunomodulatory actions and rarely occur independently. A more plausible temporal sequence is as follows: mechanical cues are first detected by peripheral mechanoreceptors and mechanotransduction systems, which then reshape microglial and astrocytic phenotypes and subsequently alter the cytokine milieu in the spinal neuroimmune microenvironment (114). This reduction in pro-inflammatory mediators-including TNF-α, IL-1β, IL-6, IL-18, ATP, and BDNF-may decrease neuronal membrane excitability and synaptic gain by limiting P2X receptor activation, attenuating TRP channel sensitization, reducing NMDA/CaMKII-dependent excitatory transmission, and preserving inhibitory signaling (115, 116). These signaling bridges allow glial modulation to influence downstream ion channel and receptor plasticity, ultimately reorganizing circuits in the spinal dorsal horn. Thus, neuroinflammatory regulation and neural plasticity should be considered sequentially linked processes rather than separate effects of mechanical stimulation within a unified mechanobiological framework. Mechanical stimulation modulates spinal sensitization via two interconnected processes: regulating peripheral nociceptive input and remodeling central neural plasticity., as shown in **Figure 4**.

### 5.1 Excitatory neurotransmission and nociceptive signaling

In chronic pain states, enhanced excitatory neurotransmission in the spinal cord is a key feature of central sensitization. Mechanical interventions reduce excitatory neurotransmitter release and block excessive nociceptive signal transmission (117). In pain models, the frequency of spontaneous excitatory postsynaptic currents (sEPSCs) in dorsal horn neurons increases, leading to heightened neuronal sensitivity. Mechanical stimulation from tuina can similarly alter sEPSCs, inhibiting or reducing excessive excitation (118). Specific excitatory interneurons, such as those expressing CGRP, are present in the dorsal horn of the spinal cord and are involved in pain signal transmission. Mechanical forces from tuina can indirectly regulate excitatory transmission by influencing the activity of these neurons (119). The excitatory-inhibitory balance within the dorsal horn network is essential for processing nociceptive information. Mechanical stimulation from tuina may induce neuroplasticity, such as enhancing inhibitory connections, thereby “shutting off” nociceptive information transmission to the brain (120). In peripheral nerve injury models, mechanical manipulation induces changes in neuroplasticity, including modulation of excitatory neurotransmission. This suggests that tuina may influence spinal cord circuits by altering synaptic strength or neuronal excitability, indirectly regulating nociceptive information processing (87). In summary, tuina weakens the transmission of nociceptive signals in the spinal cord through multiple pathways, reducing ascending stimuli and decreasing excitatory responses of postsynaptic neurons, thereby alleviating central sensitization.

### 5.2 Restoration of inhibitory systems and descending modulation

In addition to inhibiting excitatory transmission, tuina may help restore impaired inhibitory mechanisms and descending modulation systems in chronic pain (121). Chronic pain often involves dysfunction in the endogenous inhibitory pain modulation system, leading to increased excitability of pyramidal neurons in the ACC, impaired function of inhibitory interneurons, and reduced dopamine signaling. Tuina may indirectly promote the balance of inhibitory neurotransmitters by reducing dopamine release inhibition in the ACC, thereby restoring its pain modulation function (122). The imbalance between reduced descending inhibition and increased descending facilitation is a key factor in chronic pain; enhancing descending inhibition can alleviate pain (123). Tuina can indirectly regulate the endogenous opioid system and serotonin (5-HT), while enhancing descending inhibition pathways in brainstem areas, such as the locus coeruleus and raphe nuclei, thereby achieving analgesic effects (124). Overall, mechanical therapies, such as tuina, help reset the dysregulated inhibitory network. This includes enhancing local inhibitory synaptic transmission in the spinal cord and promoting the release of 5-HT, NE, and endorphins from the central descending system, counteracting the amplification of pain by central sensitization.

### 5.3 Ion channels and receptor plasticity

In chronic pain states, multiple ion channels and receptors on spinal dorsal horn neuronal membranes undergo plastic changes that facilitate neuronal excitability. The persistent activation and sensitization of nociceptors commonly observed in chronic pain syndromes are largely attributed to relief of the voltage-dependent blockade of the Piezo2 ion channel. Specifically, membrane depolarization induced by repeated noxious stimuli diminishes the voltage-dependent block of PIEZO2 channels, thereby increasing neuronal sensitivity (63). P2X receptors (P2XRs) are ligand-gated ion channels that open upon binding ATP, leading to membrane depolarization and amplification of nociceptive signal transmission. Consequently, P2XRs play a pivotal role in pathological pain conditions, including chronic pain, inflammatory diseases, and neurological disorders (125). Beyond Piezo and P2XR channels, changes in the expression and function of voltage-gated sodium and potassium channels, as well as acid-sensing ion channels (ASICs), collectively modulate nociceptive signaling (126). Mechanical stimuli, such as massage, can selectively reverse plastic changes in ion channels or receptors on spinal dorsal horn neurons, thereby indirectly modulating pain states and associated neuroplasticity. In a rat model of sciatic nerve injury, application of customized massage devices to the affected limb altered ion channel activity and reversed aberrant nociceptive perception outside the spinal dorsal horn (87). In patients with diabetic chronic pain, abdominal massage can regulate the neuro–immune–microbiota axis and thereby indirectly modify pain perception, a process that involves reversal of ion channel alterations in spinal dorsal horn neurons (127). Thus, targeting these ion channels and receptors may represent a key mechanism by which massage therapy attenuates spinal sensitization. These ion channel and receptor changes are not purely neuronal events; rather, they are shaped upstream by the neuroinflammatory milieu established by activated microglia and astrocytes.

### 5.4 Synaptic and network remodeling in the spinal dorsal horn

Under chronic pain conditions, the spinal dorsal horn, a key region for nociceptive processing, undergoes profound remodeling at the neuronal, synaptic, and network levels (128). Neural circuit plasticity includes central sensitization, synaptic plasticity, and disruption of the excitation–inhibition balance, which together result in dysfunction of the nociceptive system (129). These forms of plasticity arise from interactions between neurons and glial cells. For example, astrocytes play a pivotal role in central sensitization within the dorsal horn, particularly through chemokine receptor CXCR-mediated signaling (130). In models of chronic constriction injury–induced pain, the frequency of postsynaptic currents in dorsal horn neurons is significantly increased, reflecting enhanced synaptic transmission and a potential increase in synaptic density (118). Mechanical stimulation can remodel network function and activate dorsal horn circuits to relieve pain by modulating neuronal excitability within the dorsal horn and the endogenous opioid system (131). In addition, spinal neuromodulation techniques can reshape neural networks by stimulating the activity of specific dorsal root ganglia. This stimulation suppresses the spontaneous firing of wide dynamic range neurons in the spinal dorsal horn and reduces mechanical hypersensitivity and spontaneous pain (45). Overall, mechanical interventions such as manual therapy can relax abnormally strengthened networks and promote reorganization of spinal circuits toward a more physiological state, thereby attenuating the network basis for exaggerated pain perception.

### 5.5 Microcirculation, oxidative stress, and metabolic state

Spinal sensitization and neuroinflammation are shaped not only by synaptic and molecular alterations but also by changes in local microcirculation, oxidative stress, and energy metabolism. Chronic pain is associated with impaired local microcirculatory perfusion, tissue hypoxia, and increased oxidative stress. For example, in diabetic pain models, reduced blood flow and vascular degeneration in the dorsal horn can directly induce neuropathic pain, which manifests as behavioral pain hypersensitivity (132). Reactive oxygen species (ROS) function as signaling molecules in chronic inflammatory and neuropathic pain. They increase oxidative stress via activation of NOX2, thereby promoting dorsal root ganglion (DRG) sensitization and persistent pain (133). Peripheral injury activates the spinal AKT/mTORC1 pathway, shifting metabolism toward biosynthesis and facilitating nociceptive sensitization (134). In a model of sciatic nerve compression–induced pain, mechanical stimulation of the affected nerve increases intraneural angiogenesis and blood flow, elevates the partial pressure of oxygen within the sciatic nerve, and reduces levels of the oxidative stress marker malondialdehyde (MDA) (135). In addition, mechanical therapies can modulate systemic and local metabolism. For instance, they promote controlled ATP release followed by rapid reuptake and utilization, thereby preventing inflammation induced by excessive ATP accumulation (136). They can also improve glucose metabolism and mitigate the neurotoxic effects of a hyperglycemic environment (137). Vagal activation induced by mechanical stimulation influences hepatic glucose metabolism and adrenal stress responses, thereby attenuating the systemic hypermetabolic stress state associated with chronic pain (138). Collectively, these findings indicate that mechanical stimulation promotes restoration of normal neural function by improving oxygen and energy supply within the microenvironment and by rebalancing redox homeostasis.

## 6 Clinical and translational implications

### 6.1 Clinical evidence of mechanical stimulus in spinal-related and neuropathic pain

In clinical practice, mechanical interventions such as manual therapy are widely used to treat spine-related pain conditions, including cervical spondylosis, low back pain, sciatica, and radicular pain (see **Table 2**). Studies have shown that, compared with conventional care, manual therapy significantly improves pain and functional outcomes in patients with chronic neck pain. These benefits can persist for up to 5 weeks and 1 year after treatment (139). Moreover, combining manual therapy with dry needling or exercise therapy reduces disability in patients with neck pain, with significant improvements observed after 4 weeks of treatment (140). In spinal cord injury models, spinal cord stimulation (SCS) markedly increases mechanical pain thresholds, thereby alleviating pain hypersensitivity and reducing spine-related pain (141). Neuropathic pain is typically associated with peripheral nerve injury or inflammation. Manual therapy alleviates neuropathic pain caused by lumbar disc herniation (LDH), primarily by attenuating local neuroinflammation and thereby improving pain (85). Focal mechanical vibration, a novel form of mechanical stimulation, has been shown to be clinically feasible for the management of neuropathic pain. One report indicated that mechanical vibration significantly reduces pain, particularly in chronic neuropathic pain. It provides proprioceptive stimulation that promotes neural repair and decreases both pain intensity and duration (92). In addition, non-invasive focal repetitive trans-spinal magnetic stimulation has been shown in neuropathic pain models to upregulate Suppressor of cytokine signaling 3 (SOCS3) expression in spinal microglia, inhibit neuroinflammation, and significantly alleviate mechanical allodynia, thereby reducing pain (89). Overall, mechanical interventions such as manual therapy have a solid clinical foundation for the management of spine-related and neuropathic pain. They can effectively improve quality of life and may serve as an adjunct or alternative to neuromodulatory therapies.

**Table 2:** Clinical research on mechanical stimulation in the treatment of spinal related and neuropathic pain.

Categories	Disease	Intervention programme	Comparison method	Treatment effects	Ref.
RCT	Chronic neck pain	Tuina	Usual care	The pain score was significantly reduced and the functional recovery index was significantly improved	(139)
RCT	Non-specific chronic low back pain	Tuina	Physicotherapeutics	Pain relief, improved body function and quality of life	(142)
RCT	Chronic low back pain	Tuina	Chirismus	Reduced pain, improved quality of life, and restored lumbar function	(143)
RCT	Chronic low back pain	Tuina	Manual therapy for the spine	Short-term relief of acute and chronic low back pain, and long-term relief of chronic pain	(144)
RCT	Chronic low back pain	Tuina	Physicotherapeutics	Relieve pain, reduce disability rates, improve spinal function	(145)
RCT	neck and back pain	Tuina	No mechanical stimulation	Reduced pain intensity, reduced pain-related disability, and restored perception	(146)
RCT	Refractory painful diabetic neuropathy	High-frequency spinal cord stimulation	No mechanical stimulation	Lasting pain relief and significant improvement in heart rate and sleep	(147)
Crossover trials	Chronic neuropathic pain	Spinal cord stimulation	No mechanical stimulation	Pain reduction in CL and OL spinal cord stimulation areas	(148)
RCT	Patellofemoral pain syndrome	Manual therapy for the spine	Local motion therapy	Improvement in pain and function	(149)
RCT	Neuropathic pain	Transcutaneous electrical neural stimulation	No mechanical stimulation	Reduce pain, reduce disability	(150)
RCT	Chronic neck pain	Tuina	No mechanical stimulation	Relieve pain, reduce treatment costs	(151)
Cohort Study	Cervical spondylopathy	Tuina	No mechanical stimulation	Regulating abnormal brain activity to relieve pain and cervical dysfunction	(152)

### 6.2 Potential biomarkers linked to neuroinflammation and sensitization

To better understand and optimize the clinical effects of manual therapy, clinical studies increasingly incorporate biomarkers of neuroinflammation and spinal sensitization (see **Table 3**). SOCS3 is a potential biomarker of neuroinflammation and, when upregulated in spinal microglia, can effectively inhibit neuroinflammatory responses. Experimental studies have shown that non-invasive focal repetitive trans-spinal magnetic stimulation (rTMS) upregulates SOCS3 expression, thereby attenuating neuroinflammation and alleviating pain symptoms (89). The translocator protein (TSPO), widely regarded as a putative marker of neuroinflammation, reflects the degree of neuroinflammatory activity and is used to evaluate potential therapeutic targets. TSPO levels are elevated in neuroinflammatory states, with increased expression observed in the brain and spinal cord of patients with chronic low back pain (cLBP) (153). In addition, stimulator of interferon genes (STING) is upregulated in spinal microglia following peripheral nerve injury and is associated with neuroinflammation. It may therefore also serve as a potential biomarker (154).

**Table 3:** Candidate biomarkers of neuroinflammation and spinal sensitization.

Categories	Biomarkers	Trends	Correlation effect	Ref.
Neuroinflammation	SOCS3	down regulation	Leads to mechanical pain hypersensitivity	(89)
	TSPO	up-regulation	Exacerbate neuroinflammation	(153)
	STING	down regulation	Exacerbate neuroinflammation	(154)
	TLR4/NF-κB pathway	up-regulation	Reducing the levels of inflammatory cytokines TNF-α and IL-1β, thereby alleviating neuroinflammation	(157)
	miR-26a-5p	up-regulation	Modulation of Wnt5a/Ryk/CaMKII/NFAT signaling pathway to alleviate neuropathic pain	(158)
	Iba1	up-regulation	Increased neuroinflammation and pain behavior	(159)
	MAPKpathway	up-regulation	Leads to neuroinflammation	(160)
	P2X7receptor	up-regulation	Amplifying neuroinflammation through ATP signaling	(161)
Central sensitization	VEGF-A/NRP1pathway	up-regulation	Causes neuronal hyperexcitability and mechanical hypersensitivity	(155)
	BDNF/TrkB pathway	up-regulation	Transmit pain signals and mediate central sensitization	(156)
	NGF	up-regulation	Promoting neuron sprouting and hyperexpression of pain receptors, leading to peripheral hypersensitivity	(162)
	CX43	up-regulation	Mediates peripheral and central sensitization	(130)
	P substance	up-regulation	Increased immune reactivity drives central sensitization	(163)
	astrocyte markers	up-regulation	Together with the activation of NF-κB and NLRP3 inflammasome pathways, it exacerbates spinal cord sensitization	(164)
	TLR-4/MyD88/NF-κB pathway	up-regulation	Participates in peripheral and central sensitization with voltage-gated sodium channel (Nav1.3/1.8/1.9)	(165)
	GPX4	down regulation	Exacerbate spinal sensitization	(166)

VEGF-A is a pronociceptive factor; its interaction with NRP1 induces neuronal hyperexcitability and mechanical hypersensitivity. The VEGF-A/NRP1 pathway may therefore serve as a biomarker of neuronal sensitization (155). Moreover, BDNF/TrkB (brain-derived neurotrophic factor/tropomyosin receptor kinase B) plays a key role in the adaptation of pain transmission pathways and can serve as a biomarker of central sensitization for identifying neuropathic pain (156). In the context of neural plasticity, the NGF pathway promotes neuronal sprouting and overexpression of nociceptive pathways, leading to sensitization symptoms such as vulvar hypersensitivity. Gene signatures related to this pathway may serve as potential biomarkers of neural sensitization. Therefore, establishing objective correlations between biomarkers and the degree of pain sensitization will facilitate monitoring and optimization of the effects of mechanical therapies.

These potential biomarkers serve as key indicators of neuroinflammatory status and neural plasticity. Their expression is regulated by mechanical transduction pathways and disease-related factors, and some biomarkers are clinically used to evaluate therapeutic efficacy. Specifically, SOCS3, a negative regulator of the IL-4/JAK/STAT pathway, mediates anti-inflammatory effects and holds diagnostic and prognostic value in neuropathic pain (167). Preclinical studies show that mechanical interventions, including repetitive transspinal magnetic stimulation or postoperative exercise, can upregulate SOCS3 via upstream pathways (e.g., CaMKKβ/AMPK), thereby indirectly modulating neuroinflammation (168). However, precise force or frequency parameters have not yet been quantitatively established. TSPO primarily reflects microglial activation and mitochondrial function and serves as an imaging biomarker in neurological conditions (e.g., multiple sclerosis, epilepsy) (168). In contrast, BDNF functions as a downstream effector of IL-4/JAK/STAT signaling in Th2-driven inflammation and is modulated by specific mechanical or physical stimulation parameters, such as 10 Hz repetitive magnetic stimulation or microvibration, thereby influencing synaptic plasticity and macrophage phenotypes (169). Clinically, TSPO PET imaging reliably identifies activated neuroimmune regions and correlates with clinical subtypes (170). Collectively, these findings establish a mechanistic link among mechanical stimulation, signaling pathway modulation, biomarker expression, and clinically relevant therapeutic outcomes. They provide a framework for biomarker-informed personalized mechanotherapy.

Validating the effectiveness and reliability of biomarkers such as SOCS3, TSPO, and BDNF in mechanotherapy research requires both preclinical and clinical approaches. In preclinical studies, animal models of neuropathic or inflammatory pain can correlate biomarker expression with behavioral pain outcomes. Techniques including immunohistochemistry, ELISA, *in situ* hybridization, and single-cell RNA sequencing enable precise quantification of cytokine and glial activation profiles before and after mechanical interventions. Genetic or pharmacological manipulation of these biomarkers can further establish causal relationships between biomarker modulation and analgesic effects.

In clinical studies, stratifying patients based on baseline biomarker levels can help assess their responsiveness to mechanical interventions. Longitudinal monitoring of biomarker changes via minimally invasive sampling (blood or cerebrospinal fluid), combined with standardized pain assessments (mechanical allodynia, numeric rating scales, functional outcomes), allows evaluation of both reliability and predictive value. Integration with imaging modalities offers additional validation by linking biomarker changes to central nervous system activity. Collectively, these approaches establish the utility of these biomarkers as mechanistic indicators and predictors of treatment efficacy, thereby guiding personalized mechanical therapy protocols.

### 6.3 Integration with traditional Chinese medicine theory

Traditional Chinese Medicine (TCM) theory emphasizes the balance of the body as a whole and the activation of intrinsic self-healing mechanisms. Mechanical interventions, such as manual therapy and acupuncture, are considered means of activating this self-healing process (171). For instance, manual therapy stimulates acupoints to activate the neuro-immune axis, modulate inflammation, and restore homeostasis. This aligns with modern medical physiology and supports the TCM principle of “treating the root cause rather than merely the symptoms” (172). Concurrently, TCM meridian theory has been reinterpreted as the biochemical processes underlying mechanical stimulation. Mechanical forces applied during manual therapy deform the collagen network, activate mechanosensitive channels on mast cells, and trigger a cascade of signal transmission (173). Through biological concepts, mechanical stimulation is quantified as a process of signal initiation and propagation, providing experimental support for the practical application of TCM theory. Integrating mechanical stimulation with TCM theory helps address the operational complexity of traditional therapies, such as accurately quantifying manipulation frequency and amplitude, thereby improving therapeutic efficiency and reducing side effects. Combined with artificial intelligence and wearable devices, this integration supports the individualized treatment framework of TCM, enabling precise modulation based on multidimensional data (174). Moreover, mechanical stimulation can serve as a bridge to transform TCM practices into an evidence-based framework by elucidating external therapies and demonstrating their efficacy in hyperthermia and drug delivery (175). This expansion of TCM resources highlights its potential, especially in managing chronic diseases and pain.

Manual therapy originates from TCM, which emphasizes unblocking meridians and harmonizing qi and blood for pain relief. Modern research has provided partial explanations for these concepts: “meridian obstruction” corresponds to fascial adhesions, nerve entrapment, or circulatory disturbances in modern medicine, while “unblocking the meridians” through manual therapy can relieve fascial adhesions, release nerve entrapment, and improve local blood flow, thereby alleviating pain (176). The TCM notion of “promoting the circulation of qi and activating blood” refers to enhancing the flow of body fluids and vital energy, paralleling the improvement of tissue microcirculation and reduction of inflammation achieved by manual therapy (177). Many researchers frequently invoke TCM theory to interpret their findings. For instance, the analgesic effect of electroacupuncture is described in TCM as “harmonizing yin and yang and balancing the viscera,” corresponding, in modern terms, to the regulation of autonomic nervous function and the maintenance of endocrine and immune homeostasis (178). Similarly, the classical dictum “where there is pain, there is no free flow; where there is no free flow, there is pain” aligns with the modern understanding that chronic pain is often associated with tissue adhesions and impaired conduction pathways. Manual therapy alleviates pain by restoring tissue permeability and conduction (179). These traditional concepts guide clinical practice and provide valuable insights for research.

### 6.4 Methodological considerations and standardization of mechanical parameters

Although research on the mechanisms of mechanical stimulation is increasing, methodological heterogeneity in both clinical and experimental studies significantly limits the comparability and meta-analysis of results. First, the standardization of intervention parameters remains an issue. Inconsistent or incomplete reporting of factors such as force, frequency, and duration in manual therapy or acupuncture makes comparison and replication of studies difficult (180). For instance, some electroacupuncture treatments use 2Hz/100Hz sparse-dense wave stimulation (181), while others use continuous electrical stimulation (182). However, the literature often lacks detailed quantification of parameters, such as current intensity. Manual therapy lacks objective, quantitative indicators and is primarily based on the practitioner’s experience. The lack of studies on the mechanical dose-response relationship makes it difficult to determine the optimal intensity and duration of manual therapy. Future research should focus on improving standardization in both animal experiments and clinical trials, such as using pressure sensors and motion capture systems to record the details of manual techniques. Secondly, randomization and blinding are challenging to implement in manual therapy studies. Practitioners can easily distinguish between manual therapy and sham interventions, and differences in techniques among practitioners may influence efficacy. Some studies use “light touch” as a control, but this can still lead to therapeutic effects (183). Additionally, there are varying standards for outcome measures across studies, with some focusing on pain scores and others on biological markers. These methodological issues highlight the need for caution in interpreting results and stress the importance of more rigorous and standardized designs in future research. In conclusion, establishing standardized protocols for mechanical stimulation research, including intervention parameters, control settings, blinding procedures, and outcome selection, is crucial for drawing reliable conclusions and advancing clinical applications.

## 7 Future directions

### 7.1 Key knowledge gaps in mechanobiology of mechanical stimulus

Although evidence suggests that mechanical stimulation through manual therapy can regulate neuroinflammation and spinal sensitization, the underlying biomechanical mechanisms remain unclear. Several unresolved questions need further investigation: How are mechanical stimuli sensed and transmitted at the molecular level? Although some mechanosensitive channels are known, our understanding of how mechanical signals are converted within glial cells and neurons remains limited (184). For instance, the role of integrin-cytoskeleton signaling complexes in the spinal cord is still unclear. Moreover, the specific effects of different mechanical stimuli, such as pressure, stretching, and vibration, on neuroimmune cells should be systematically compared (185). It remains unclear which components of manual therapy are most crucial for anti-inflammatory and analgesic effects. Furthermore, how individual factors such as age, sex, and genetic background influence the efficacy of manual therapy is still unknown (186). Some patients respond well to manual therapy, while others show limited improvement. The biological reasons for this variability warrant further exploration. These knowledge gaps highlight important directions for future research.

### 7.2 Advanced technologies and multi-omics approaches

Cutting-edge technologies will provide strong support for understanding the mechanisms of mechanical stimulation. First, optogenetics and chemogenetics can precisely control the activity of specific sensory neurons or glial cells, simulating or blocking mechanical signal pathways to identify key processes (187). Second, single-cell omics (e.g., single-cell RNA sequencing and mass spectrometry proteomics) can analyze gene expression changes in different cell types in the spinal cord and DRG following mechanical interventions, identifying specific molecular pathways (188). For example, single-cell sequencing could identify unique markers for “mechanical stimulus-responsive cells.” Third, high-resolution imaging techniques, like two-photon microscopy, can observe *in vivo* dynamics of glial cell-neuron interactions under mechanical forces in real time, offering direct insight into the mechanical effects (189). Machine learning and computational modeling can integrate multi-omics and imaging data to build predictive models of the relationship between mechanical stimulation, inflammation, and pain behavior, helping select the most effective intervention parameters (190). Overall, integrating these new technologies into traditional manual therapy research will greatly enhance our understanding of its mechanisms, making it more precise and scientific.

### 7.3 Development of standardized and individualized mechanical stimulation protocols

While research on mechanisms progresses, developing mechanical stimulation protocols that are both standardized and adaptable to individual needs is equally crucial. Standardization requires defining best practices, including establishing manual therapy guidelines for various diseases, such as specifying treatment locations, force, and frequency, and developing objective efficacy evaluation systems. This would allow manual therapy to follow a clear “instruction manual,” similar to pharmaceuticals (12). Achieving this may require large-scale clinical studies, such as dose-response trials to identify effective pressure ranges and treatment frequencies. Individualization is equally important, as patients’ conditions and constitutions vary significantly. Ideal mechanical therapy should adjust protocols based on factors such as the degree of sensitization, inflammation levels, and comorbidities. For example, in cases with significant inflammation, the focus should be on anti-inflammatory techniques, while in cases with pronounced sensitization, sedative and muscle-relaxing techniques should take precedence. Real-time biofeedback technologies could enable practitioners to dynamically adjust treatment force based on changes in muscle tone and skin temperature (191). In the future, artificial intelligence could predict the most suitable therapy combinations based on patient data, enabling true individualized treatment. In conclusion, combining standardization and individualization will be the hallmark of the maturation of mechanical therapy.

### 7.4 Toward integrative and personalized pain management

Pain onset and relief result from a variety of factors. From a broader perspective, manual therapy should be considered a key component of an integrated and personalized pain management system. Mechanical therapies, such as manual therapy, can be combined with pharmacological treatments, psychological counseling, and physical therapies to create a comprehensive intervention strategy. For example, for patients with chronic low back pain, combining manual therapy and exercise rehabilitation with pharmacological treatment has been shown to produce better and more lasting effects than monotherapy (192). Integrating mechanical therapy also calls for a reevaluation of pain assessment, which should not only focus on pain intensity scores but also consider inflammatory markers, functional status, and psychological stress to comprehensively assess treatment efficacy. Finally, as non-pharmacological therapies gain acceptance, manual therapy holds greater potential for managing chronic pain and opioid substitution (193) and could become a key component of chronic disease management and community healthcare. It is foreseeable that, in future pain management systems, individualized mechanical stimulation protocols, combined with other therapies, will offer patients safer, more efficient, and comprehensive pain relief strategies.

Personalized mechanical interventions can improve therapeutic efficacy by adjusting stimulation parameters according to individual patient characteristics. For example, patients with elevated pro-inflammatory cytokines (TNF-α, IL-1β, IL-6) may benefit from low-frequency, prolonged mechanical stimulation to modulate glial activation and reduce cytokine release. In contrast, patients with moderate inflammation may tolerate higher-frequency interventions that enhance tissue perfusion and mechanotransduction (194). Genetic polymorphisms in mechanosensitive ion channels (Piezo2, TRPV4) or cytokine receptors may further guide the selection of stimulus type, intensity, and duration (195). Integrating real-time physiological feedback, such as muscle activity, skin perfusion, or EMG signals, may enable dynamic adjustment of mechanical parameters to optimize analgesic and anti-inflammatory effects. Collectively, these approaches provide a framework for precision mechanotherapy, connecting mechanobiological interventions to patient-specific neuroimmune and genetic profiles.

**Figure 5:**
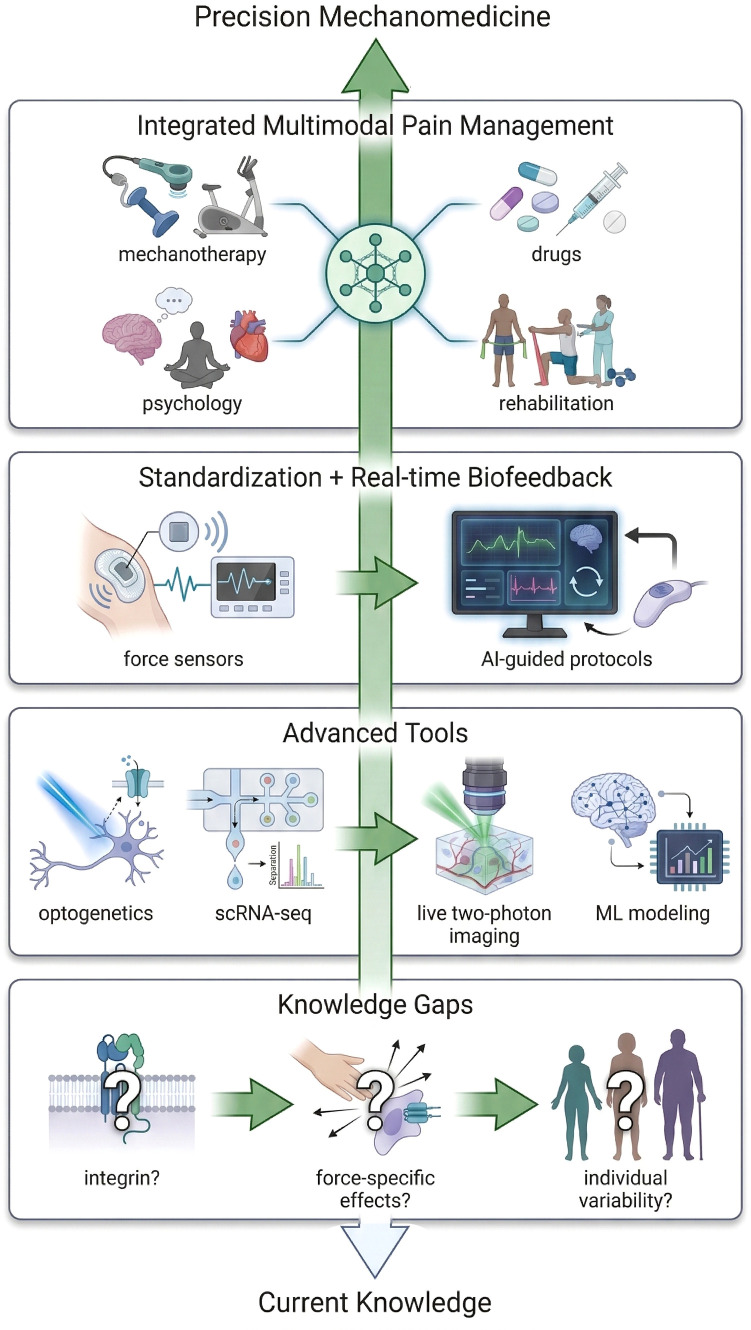
The transformation path of massage analgesia with biomechanics as the core: from mechanism analysis to individualized pain management.

Future research will focus on the logical sequence of “precisely understanding mechanisms, targeting regulatory networks, and clinical translation,” promoting the transition of mechanical stimulation from “basic research” to “new strategies for chronic pain treatment” through technological innovation and interdisciplinary collaboration, and providing a “non-pharmacological, individualized” approach to chronic pain treatment (**Figure 5**).

## 8 Conclusion

Neuroinflammation-driven spinal sensitization is the core mechanism behind chronic pain persistence. Mechanical stimulation, such as manual therapy, intervenes in the neuro-immune-mechanical network, providing multi-level regulation of this process. Extensive basic research shows that moderate mechanical forces inhibit glial cell overactivation, restore the balance of inflammatory factors, and re-establish excitatory-inhibitory homeostasis in the spinal cord, thereby reversing central sensitization. Mechanical interventions, such as manual therapy, provide a mechanism-based foundation for non-pharmacological analgesia. Their academic value lies in revealing the mechanobiological principles of pain regulation and promoting the quantification and reproducibility of traditional therapies. As a safe and adjustable intervention, mechanical stimulation offers significant clinical and public health potential in chronic pain management. Future research should focus on multi-omics and standardized methods to establish individualized mechanical intervention models, offering a systematic and precise approach for chronic pain prevention and treatment.
